# Association of Retinol and Carotenoids Content in Diet and Serum With Risk for Colorectal Cancer: A Meta-Analysis

**DOI:** 10.3389/fnut.2022.918777

**Published:** 2022-06-30

**Authors:** Xiaoyong Han, Rangyin Zhao, Guangming Zhang, Yajun Jiao, Yongfeng Wang, Da Wang, Hui Cai

**Affiliations:** ^1^Graduate School, Ning Xia Medical University, Yinchuan, China; ^2^General Surgery Clinical Medical Center, Gansu Provincial Hospital, Lanzhou, China; ^3^Key Laboratory of Molecular Diagnostics and Precision Medicine for Surgical Oncology in Gansu Province, Gansu Provincial Hospital, Lanzhou, China; ^4^First Clinical Medical College, Gansu University of Chinese Medicine, Lanzhou, China; ^5^Medical College of Jiangsu University, Zhenjiang, China; ^6^First Clinical College of Medicine, Lanzhou University, Lanzhou, China; ^7^NHC Key Laboratory of Diagnosis and Therapy of Gastrointestinal Tumor, Gansu Provincial Hospital, Lanzhou, China

**Keywords:** retinol, carotenoids, colorectal cancer, risk, meta-analysis

## Abstract

**Background:**

Colorectal cancer (CRC) risk is linked to serum and dietary retinol and carotenoids, according to clinical and epidemiological research. However, the findings are not consistent. As a result, we did this meta-analysis to determine the link between them.

**Methods:**

From 2000 through 2022, the PubMed, Web of Science, and Embase databases, as well as pertinent article references, were searched and filtered based on inclusion and exclusion criteria and literature quality ratings. High and low intake were used as controls, and OR (odds ratio) or RR (relative risk) and 95% confidence interval were extracted. The extracted data were plotted and analyzed using Stata12.0 software.

**Results:**

A total of 22 relevant studies were included, including 18 studies related to diet and 4 studies related to serum. For high and low intake or concentration controls, the pooled OR was as follows: β-carotene (OR = 0.89, 95% CI: 0.78–1.03), α-carotene (OR = 0.87, 95% CI: 0.72–1.03), lycopene (OR = 0.93, 95% CI: 0.81–1.07), lutein/zeaxanthin (OR = 0.96, 95% CI: 0.87–1.07), β-cryptoxanthin (OR = 0.70, 95% CI: 0.48–1.01), total carotenoids (OR = 0.97, 95% CI: 0.81–1.15), retinol (OR = 0.99, 95% CI: 0.89–1.10), serum carotenoids (OR = 0.73, 95% CI: 0.58–0.93), serum retinol (OR = 0.62, 95% CI: 0.26–1.49). Subgroup analysis was performed according to tumor type, study type and sex.

**Conclusion:**

Total carotenoid intake and Lutein/Zeaxanthin intake were not associated with CRC risk. High β-carotene, α-carotene, lycopene, and β-cryptoxanthin all tended to reduce CRC risk. Serum carotenoid concentrations were significantly inversely associated with CRC risk.

## Introduction

In recent years, the incidence and mortality of malignant tumors have been increasing year by year, even exceeding other chronic diseases, becoming a veritable human health killer (1). Although the efficacy of cancer treatment has been improved due to comprehensive therapies such as surgery, chemotherapy, radiotherapy, targeted therapy, and immunotherapy, the prognosis and early diagnosis remain poor and the mortality rate remains high (2). Colorectal cancer (CRC) is the world’s third most frequent cancer and the second largest cause of cancer mortality, with a significant number of new cases and deaths every year (3). CRC has caused great burden and harm to the economy and society of the country (4). Economic development and changes in lifestyle and dietary choices have increased the prevalence and mortality of CRC in China in recent years, putting a strain on the health-care system (3, 5). The etiology of CRC is heavily influenced by environmental and genetic factors. Diet, history of benign adenomatous polyps and inflammatory bowel disease, age, diabetes, obesity, lack of physical activity, and a family history of CRC are all risk factors for CRC (6). Therefore, the prevention of CRC by changing dietary habits and lifestyle is an area that we should focus on.

Fruits and vegetables are among the daily foods required for good health since they include high levels of minerals, vitamins, carbs, proteins, dietary fiber, and different substances with nutritional medicinal value that can help prevent a variety of ailments (7). Many studies have indicated that eating fruits and vegetables helps prevent cancer, with vegetable-related protection being more substantial (8, 9). Vitamin A is an unsaturated hydrocarbon group that includes retinol and its derivatives such as retinaldehyde, retinoic acid, and retinyl ester (10). Cell development and differentiation, embryogenesis, reproduction, epithelial cell integrity, and immunological function are all regulated by vitamin A (11, 12). It also has antioxidant properties (13) and helps to reduce oxidative stress damage and inflammation (11, 14). Carotenoids are a good source of vitamin A and may be turned into it by the body (15). Carotenoids are natural pigments found in a wide range of fruits and vegetables, including lycopene, β-carotene, lutein, zeaxanthin, and β-cryptoxanthin (16). Carotenoids and retinoids share many biological actions, including antioxidant capabilities, suppression of malignant tumor development, and activation of apoptosis (17). In addition, carotenoids can influence cell development, as well as gene expression and immunological responses (18, 19). Thus, retinol and carotenoids are indispensable in the human body. But retinol cannot be synthesized in the human body, and it must be obtained from the diet (20). As a result, research into the relationship between their consumption and human illnesses, including cancer, is required.

Over the last two decades, researchers have conducted substantial research on the link between nutrition and cancer. Epidemiological studies have found a link between food and cancer incidence and aggressiveness (21). A high intake of dietary carotenoids or vitamin A (retinol) has been linked to a decreased risk of CRC in several studies (22–25). However, other studies have shown no substantial link between their use and the risk of cancer onset (25–27). In addition to diet, there has been interest in the research of serum retinol and carotenoids, and some studies have shown that their levels in the blood are related to the risk of colon cancer. As a result, we completed our meta-analysis in time to incorporate the most recent relevant data, providing more credible scientific support for CRC prevention.

## Materials and Methods

### Search Strategy for Literature

Two writers (Xiaoyong Han and Rangyin Zhao) separately conducted a literature search for the association between retinol, carotenoids, and related derivatives and the risk of CRC in humans using the PubMed, Web of Science and Embase databases. The following keywords were used in the search: “retinol” or “carotenoids” or “carotene” or α-carotene or “β-carotene” or “cryptoxanthin” or “lycopene” or “lutein” or “zeaxanthin” combined with “colorectal cancer” or “colon cancer” or “rectal cancer.” All relevant literature was searched from 2000 to April 2022. In addition, we performed a manual search of the reference lists of reviews, meta-analyses, and other relevant publications to prevent potentially missed articles. The language of included articles was limited to English.

### Inclusion and Exclusion Criteria

Studies were included according to the following criteria: (1) patients were diagnosed with colon or rectal cancer; (2) observational studies, including cohort or case-control studies; (3) The associations of interest are about the association of serum or dietary retinol or carotenoids with CRC risk, and there are comparisons of high and low content.; (4) Included studies contained relative risks (RR) or odds ratios (OR) with 95% confidence intervals for CRC. The following exclusion criteria were used: (1) reviews or conferences or abstracts or letters to the editor; (2) duplicate study populations; (3) animal studies; (4) other cancer studies; (5) lack of RR or OR data; (6) other vitamin supplement studies.

### Data Extraction and Quality Evaluation

All included papers were examined and relevant data were retrieved independently by two researchers. Inclusion basic information included: name of first author, date of publication, country, type of study, vitamin type, cancer type, sample size of cases and controls, RR or OR and 95% CI for cancer, covariate correction. The disagreement between these two researchers was decided jointly by a third author. The quality of the included studies was assessed using the NOS scoring criteria (0–9 points), and those with a score > 6 were included in the meta-analysis.

### Statistical Analysis

RR or OR with 95% confidence intervals were extracted from each study to assess the association of high retinol or carotenoids intake with cancer risk. The results generally combined in cohort studies are RR values, and the results generally combined in case-control studies are OR values. In order to better calculate and combine the results of studies, the difference between the two is negligible, and all the results are expressed as OR values. In addition, heterogeneity among studies was assessed by *Q*-test and *I*^2^ statistic. *Q*-test (*P*_*Q*_) *p*-value < 0.1 and *I*^2^ > 50% indicated that there was significant statistical heterogeneity between studies, and the results were analyzed using a random-effects model. Otherwise, a fixed effects model was used. We used forest plots to present the meta-analysis results and used Begg’s test as well as Begg’s funnel plots to assess publication bias. In addition, by eliminating each study one by one, a sensitivity analysis was performed to check the stability of the results. Analyses were performed using Stata12.0 for Windows (Stata, College Station, TX, United States) and *p* < 0.05 was considered statistically significant.

## Results

### Screening Process for Eligible Literatures

**FIGURE 1:**
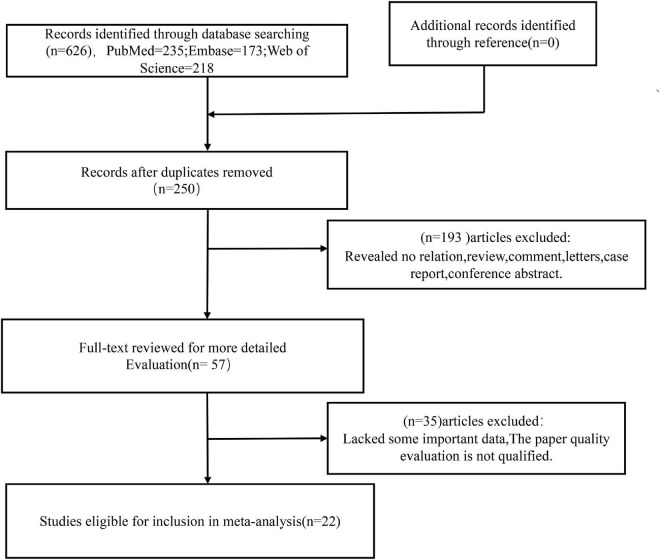
Flow diagram of this meta-analysis.

The relevant literatures were searched in three main English databases according to the search strategy: PubMed (*n* = 235), Web of Science (*n* = 218), Embase (*n* = 173). After de-duplication (*n* = 376), the titles and abstracts of the remaining articles (*n* = 250) were examined and evaluated. A total of 193 articles were rejected for purpose, article type (review, case study, or conference abstract), or irrelevant findings. Fifty-seven full-text articles were downloaded, of which 35 studies were rejected after initial analysis due to lack of important data or unsatisfactory quality of NOS scores. Finally, the meta-analysis comprised 22 papers that fully fulfilled the inclusion criteria and quality evaluation. Figure 1 depicts the search flow chart.

### Characteristics of Included Research Projects

Table 1 shows the main characteristics of the 22 included studies. Regarding dietary aspects, a total of five cohort studies were included, and 399,558 individuals were followed up for 5–15 years, eventually resulting in 6,919 CRC patients. A total of 13 case-control studies involving 11,029 cases and 19,024 controls were included. With respect to serum, two cohort studies were included, with 32,428 participants and, ultimately, 272 patients with CRC. Two case-control studies involving 1,073 cases and 1,116 controls were included. Studies were published between 2000 and 2019. Eight studies were from European countries, eight from North American countries and six from Asian countries. The major nutrient species studied were carotenoids, lycopene, α-carotene, β-carotene, lutein/zeaxanthin, β-cryptoxanthin, and retinol. The included studies were adjusted for covariates, mainly including: gender, age, smoking, alcohol consumption, family history of CRC, and physical activity. The NOS was scored from 6 to 8. The original data of the included studies are found in Supplementary Table S1.

**TABLE 1:** Characteristics of included studies.

References Country	Type of cancer	Type of study	Sample size	Diet/Serum	Nutrient type	Adjustment for covariates.	NOS score
Roswall et al. (27) Denmark	Colon and rectal cancer	Cohort study	56,332/748	Diet	β-carotene	Education, alcohol consumption, consumption of red and processed meat, smoking status	7
Murtaugh et al. (23) United States	Rectal cancer	Case-control study	952/1,205	Diet	Lycopene, β-carotene, lutein	Age, body mass index, physical activity, energy intake, dietary fiber, dietary calcium, and smoking status	7
Williams et al. (53) United States	Colorectal cancer	Case-control study	945/959	Diet	β-carotene	Age, gender, education, smoking status, BMI, physical activity, family history, history of alcohol use	6
Park et al. (26) United States	Colon and rectal cancer	Cohort study	191,004/2,378	Diet	Lycopene, α-carotene, β-carotene, carotenoids, β-cryptoxanthin, Lutein	Gender, age, family history of colorectal cancer, history of intestinal polyps, number of pack-years smoked, body mass index	8
Slattery et al. (54) United States	Colon cancer	Case-control study	1,993/2,410	Diet	Lycopene, α-carotene, β-carotene, β-cryptoxanthin, Lutein, zeaxanthin	Age, gender, smoking, alcohol consumption, BMI and long term strenuous physical activity	7
Leenders et al. (22) Europe	Colon and rectal cancer	Case-control study	1,399/1,399	Diet	Lycopene, α-carotene, β-carotene, carotenoids, retinol	Smoking, alcohol consumption, BMI, physical activity, consumption level	7
Terry et al. (55) Canada	Colon and rectal cancer	Cohort study	56,837/5,681	Diet	Lycopene, α-carotene, β-carotene, carotenoids	Smoking status, relative body mass (body mass index), total fat intake, energy, alcohol, and folic acid, or menopausal status	7
Nkondjock and Ghadirian (56) Canada	Colon cancer	Case-control study	402/688	Diet	Lycopene, α-carotene, β-carotene, carotenoids, lutein/zeaxanthin, β-cryptoxanthin	Age, history of CC in first-degree relatives, marital status, gender, physical activity, fiber and folate consumption, and total energy intake	7
Wang et al. (57) Japan	Colon and rectal cancer	Case-control study	816/815	Diet	Lycopene, carotenoids	Age, residence, family history of colorectal cancer, smoking, alcohol consumption, BMI, type of work, physical activity	6
Negri et al. (58) Italy	Colorectal cancer	Case-control study	1,953/4,154	Diet	Lycopene, carotenoids, retinol	Sociodemographic characteristics, smoking, physical activity, anthropometric measurements at different ages, family history of cancer	7
Levi et al. (59) Switzerland	Colorectal cancer	Case-control study	223/491	Diet	Carotenoids, retinol	Age, sex, education, smoking, alcohol, body mass index, physical activity, and total energy and fiber intake	7
Lu et al. (60) China	Colorectal cancer	Case-control study	845/845	Diet	Lycopene, α-carotene, β-carotene, carotenoids, lutein/zeaxanthin, β-cryptoxanthin	Education, marital status, occupation, income, family history of cancer, smoking status, passive smoking, alcohol consumption, occupational activities, family and leisure activities, BMI	7
Paiva et al. (61) Portugal	Colorectal cancer	Case-control study	100/211	Diet	Carotenoids	Age, sex, marital status, work physical activity, family history of cancer, body mass index, fiber, carotene, vitamin C, and total energy	7
Rosato et al. (24) Switzerland	Colorectal cancer	Case-control study	329/1,361	Diet	β-carotene	Age, gender, family history, alcohol use, education, physical activity	6
Key et al. (62) United Kingdom	Colorectal cancer	Case-control study	565/1,951	Diet	β-carotene	Height, weight, energy intake, alcohol intake, dietary fiber, smoking, alcohol	
						consumption, physical activity, education, social class	7
Cook et al. (63) United States	Colon and rectal cancer	Cohort study	22,071/267	Diet	β-carotene	Age, education, marital status, occupation, income, family history of cancer, smoking status, passive smoking, alcohol consumption, occupational activity, BMI	7
Wakai et al. (64) Japan	Colon and rectal cancer	Case-control study	507/2,535	Diet	Carotenoids, retinol	Sex, age, family history, smoking, alcohol use, physical activity, energy intake	7
Shin et al. (25) China	Colon and rectal cancer	Cohort study	73,314/283	Diet	Carotenoids, retinol	Age, menopausal status, education, smoking, alcohol consumption, physical activity, family history of colorectal cancer, use of vitamin supplements, and total energy intake	8
Kabat et al. (65) United States	Colorectal cancer	Cohort study	5,477/88	Serum	Lycopene,α-carotene, β-carotene, Lutein + Zeaxanthin,β-Cryptoxanthin, Retinol	Age, body mass index, waist circumference, alcohol intake, physical activity, family history of colorectal cancer, ethnicity	8
Huang et al. (28) China	Colorectal cancer	Case-control study	538/564	Serum	Lycopene, α-carotene, β-carotene, lutein/zeaxanthin, β-cryptoxanthin	Living conditions, educational level, occupation, income, study, alcohol consumption, family history of colorectal cancer, physical activity	7
Luo et al. (66) China	Colon and rectal cancer	Case-control study	535/552	Serum	Retinol	Age, sex, residence, educational level, marital status, income, family and leisure activities, passive smoking, alcohol consumption, adult height, and BMI	6
Malila et al. (67) Finland	Colorectal cancer	Cohort study	26,951/184	Serum	Retinol, β-carotene	Age, body mass index (BMI), number of cigarettes smoked per day, occupational and leisure time physical activity, serum cholesterol concentration, alcohol intake	8

### Association Between Dietary Retinol and Various Carotenoids and Colorectal Cancer Risk

#### β-Carotene

**FIGURE 2:**
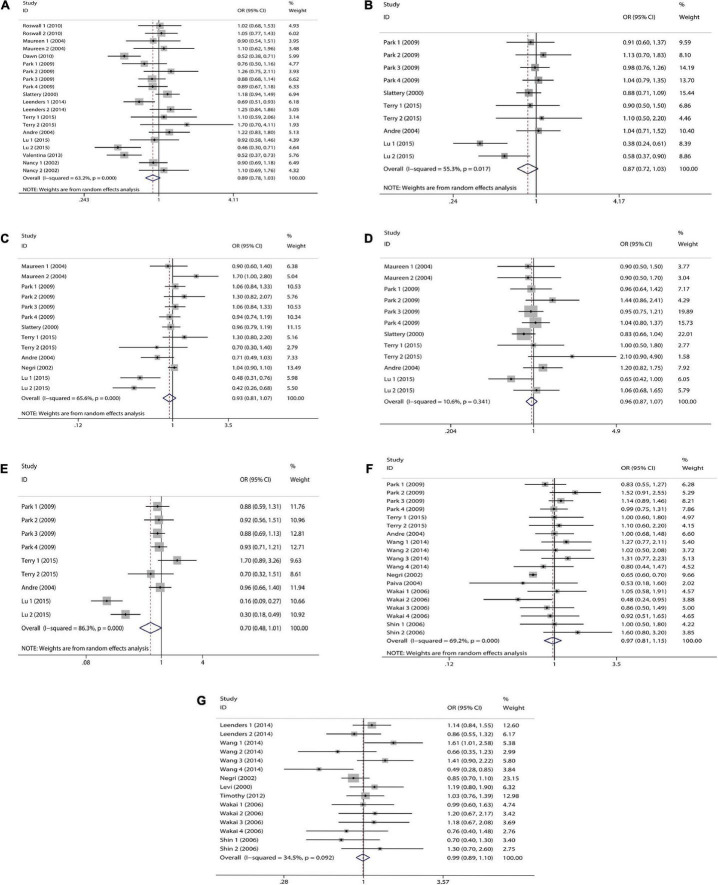
Forest plot on dietary intake of carotenoids and retinol and colorectal cancer risk. **(A)** β-carotene; **(B)** α-carotene; **(C)** lycopene; **(D)** lutein/zeaxanthin; **(E)** β-Cryptoxanthin; **(F)** carotenoids; **(G)** retinol.

**FIGURE 3:**
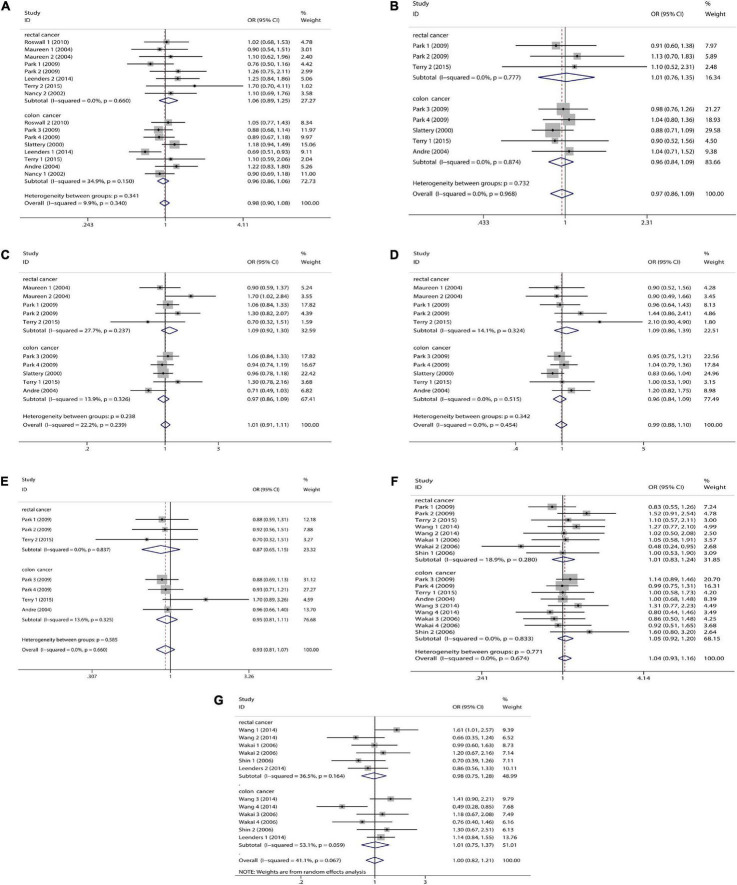
Tumor subgroup analysis of dietary carotenoid and retinol intake and colorectal cancer risk. **(A)** β-carotene; **(B)** α-carotene; **(C)** lycopene; **(D)** lutein/zeaxanthin; **(E)** β-Cryptoxanthin; **(F)** carotenoids; **(G)** retinol.

**FIGURE 4:**
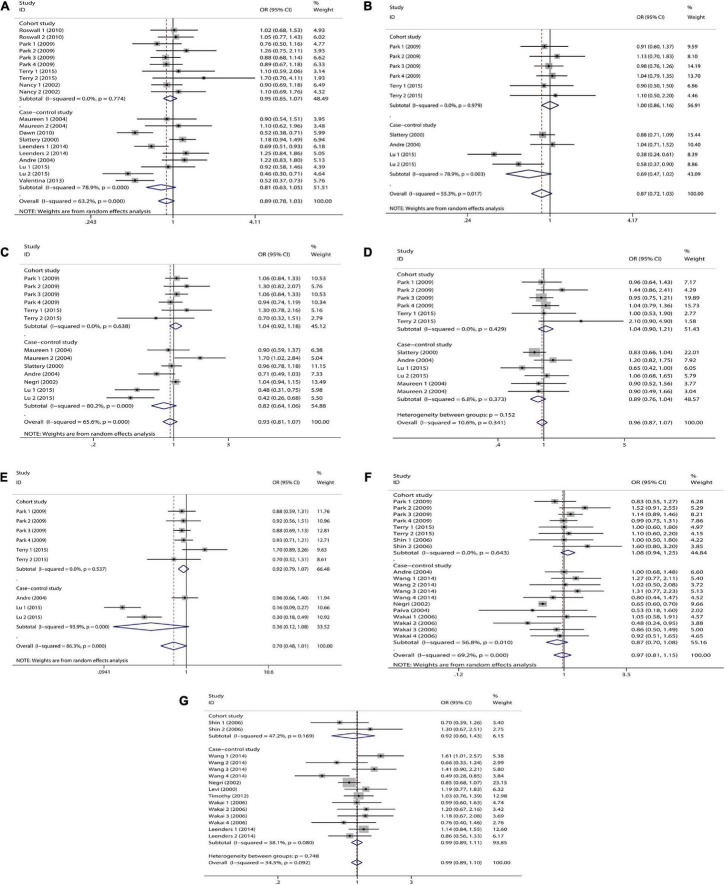
Study type subgroup analysis of dietary carotenoid and retinol intake and colorectal cancer risk. **(A)** β-carotene; **(B)** α-carotene; **(C)** lycopene; **(D)** lutein/zeaxanthin; **(E)** β-Cryptoxanthin; **(F)** carotenoids; **(G)** retinol.

**FIGURE 5:**
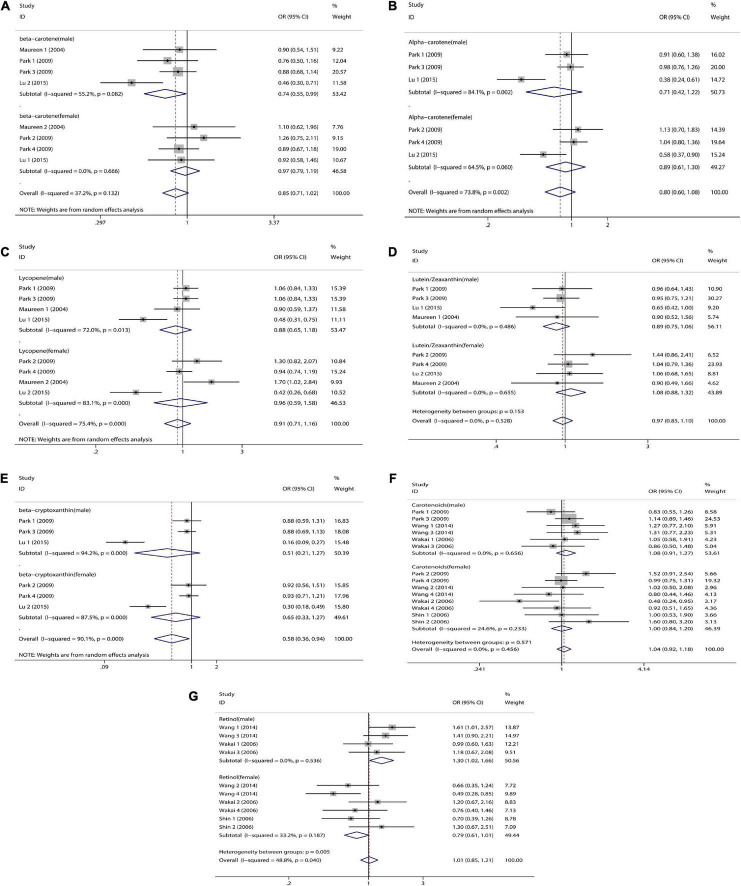
Sex subgroup analysis of dietary carotenoid and retinol intake and colorectal cancer risk. **(A)** β-carotene; **(B)** α-carotene; **(C)** lycopene; **(D)** lutein/zeaxanthin; **(E)** β-Cryptoxanthin; **(F)** carotenoids; **(G)** retinol.

We combined a total of 20 sets of data from 11 studies. Comparing low intakes, dietary high intake of β-carotene reduced the risk of CRC by 11% (OR = 0.89, 95% CI: 0.78–1.03, Figure 2A), but the association between the two was not significant (*p_*t*_* = 0.113), and due to significant heterogeneity (*I*^2^ = 63.2%, *p* < 0.001), we used a random-effects model for pooled analysis. According to subgroup analysis by tumor type, it can be seen that there is no significant correlation between dietary intake and the risk of colon cancer (OR = 0.96, 95% CI: 0.86–1.06, Figure 3A) and rectal cancer (OR = 1.06, 95% CI: 0.89–1.25, Figure 3A). In the subgroup analysis by study type, both the cohort study (OR = 0.95, 95% CI: 0.85–1.07, Figure 4A) and the case-control study (OR = 0.81, 95% CI: 0.63–1.05, Figure 4A) showed a trend of β-carotene to reduce the risk of CRC, but none of them were significantly associated. Finally, according to gender subgroup analysis, β-carotene intake was not significantly associated with the risk of CRC in female (OR = 0.97, 95% CI: 0.79–1.19, Figure 5A), but β-carotene intake was negatively associated with the risk of CRC in male (OR = 0.74, 95% CI: 0.55–0.99, Figure 5A).

#### α-Carotene

We combined a total of 10 sets of data from 5 studies. Comparing low intakes, dietary high intake of α-carotene reduced the risk of CRC by 13% (OR = 0.87, 95% CI: 0.72–1.03, Figure 2B), but the association between the two was not significant (*p_*t*_* = 0.110), and due to significant heterogeneity (*I*^2^ = 55.3%, *p* = 0.017), we used a random-effects model for pooled analysis. Subgroup analysis by tumor type showed that dietary intake was not significantly associated with the risk of colon cancer (OR = 0.96, 95% CI: 0.84–1.09, Figure 3B) and rectal cancer (OR = 1.01, 95% CI: 0.76–1.35, Figure 3B). In the subgroup analysis by study type, the cohort study (OR = 1.00, 95% CI: 0.86–1.16, Figure 4B) showed no significant association between their intake and CRC, and the case-control studies (OR = 0.69, 95% CI: 0.47–1.02, Figure 4B) showed that their high intake tended to reduce CRC risk, but there was no significant association. Finally, high intake of α-carotene tended to reduce CRC in male (OR = 0.71, 95% CI: 0.42–1.22, Figure 5B) and female (OR = 0.89, 95% CI: 0.61–1.30, Figure 5B) according to gender subgroup analysis, but there was no significant association.

#### Lycopene

Seven studies were included to combine a total of 13 sets of data. High lycopene (OR = 0.93, 95% CI: 0.81–1.07, Figure 2C) intake slightly, but not significantly (*p_*t*_* = 0.329), reduced CRC risk. Due to significant heterogeneity (*I*^2^ = 65.6%, *p* = 0.000), pooling was performed with a random-effects model. Subgroup analysis was performed according to tumor type, study type and gender. Colon cancer (OR = 0.97, 95% CI: 0.86–1.09, Figure 3C), rectal cancer (OR = 1.09, 95% CI: 0.92–1.30, Figure 3C), cohort study (OR = 1.04, 95% CI: 0.92–1.18, Figure 4C), case-control study (OR = 0.82, 95% CI: 0.64–1.06, Figure 4C), male (OR = 0.88, 95% CI: 0.65–1.18, Figure 5C), female (OR = 0.96, 95% CI: 0.59–1.58, Figure 5C). In subgroup analyses, case-control studies showed a non-significant inverse association between lycopene intake and CRC risk. There was also a risk reduction effect in male, although it was not significant.

#### Lutein/Zeaxanthin

Six studies were included and a total of 12 sets of data were combined. There was no significant (*p_*t*_* = 0.508) association between high lutein/zeaxanthin (OR = 0.96, 95% CI: 0.87–1.07, Figure 2D) intake and CRC risk. No significant heterogeneity was found (*I*^2^ = 10.6%, *p* = 0.341), which was summarized using a fixed-effect model. Subgroup analysis was performed according to tumor type, study type and gender. Colon cancer (OR = 0.96, 95% CI: 0.84–1.09, Figure 3D), rectal cancer (OR = 1.09, 95% CI: 0.86–1.39, Figure 3D), cohort study (OR = 1.04, 95% CI: 0.90–1.21, Figure 4D), case-control study (OR = 0.89, 95% CI: 0.76–1.04, Figure 4D), male (OR = 0.89, 95% CI: 0.75–1.06, Figure 5D), female (OR = 1.08, 95% CI: 0.88–1.32, Figure 5D). In subgroup analysis, case-control studies showed a non-significant inverse association between the intake of lutein/zeaxanthin and CRC risk. The risk reduction effect was also present in male, but was not significant.

#### β-Cryptoxanthin

Four studies were included and a total of 9 sets of data were combined. High β-cryptoxanthin (OR = 0.70, 95% CI: 0.48–1.01, Figure 2E) intake was able to reduce CRC risk by 30%, but not statistically significant (*p_*t*_* = 0.058). High heterogeneity was found (*I*^2^ = 86.3%, *p* = 0.000), which was combined using the random-effects model. Subgroup analysis was performed according to tumor type, study type, and gender. Colon cancer (OR = 0.95, 95% CI: 0.81–1.11, Figure 3E), rectal cancer (OR = 0.87, 95% CI: 0.65–1.15, Figure 3E), cohort study (OR = 0.92, 95% CI: 0.79–1.07, Figure 4E), case-control study (OR = 0.36, 95% CI: 0.12–1.08, Figure 4E), male (OR = 0.51, 95% CI: 0.21–1.27, Figure 5E), female (OR = 0.65, 95% CI: 0.33–1.27, Figure 5E). In subgroup analysis, high β-cryptoxanthin intake tended to decrease risk of CRC, but this was not significant.

#### Total Carotenoids

Eight studies were included and a total of 19 sets of data were combined. There was no significant (*p_*t*_* = 0.717) association between high carotenoids (OR = 0.97, 95% CI: 0.81–1.15, Figure 2F) intake and CRC risk. There was significant heterogeneity (*I*^2^ = 69.2%, *p* = 0.000), which was combined using the random-effects model. Subgroup analysis was performed according to tumor type, study type and gender. Colon cancer (OR = 1.05, 95% CI: 0.92–1.20, Figure 3F), rectal cancer (OR = 1.01, 95% CI: 0.83–1.24, Figure 3F), cohort study (OR = 1.08, 95% CI: 0.94–1.25, Figure 4F), case-control study (OR = 0.87, 95% CI: 0.70–1.08, Figure 4F), male (OR = 1.08, 95% CI: 0.91–1.27, Figure 5F), female (OR = 1.00, 95% CI: 0.84–1.20, Figure 5F). No association was found between high carotenoids intake and the risk of CRC in any Subgroup group.

#### Retinol

Seven studies were included and a total of 15 sets of data were combined. There was no significant (*p*_*t*_ = 0.850) association between high retinol (OR = 0.99, 95% CI: 0.89–1.10, Figure 2G) intake and CRC risk. There was no significant heterogeneity (*I*^2^ = 34.5%, *p* = 0.092), and fixed effect model was used for combination. Subgroup analysis was performed according to tumor type, study type and gender. Colon cancer (OR = 1.01, 95% CI: 0.75–1.37, Figure 3G), rectal cancer (OR = 0.98, 95% CI: 0.75–1.28, Figure 3G), cohort study (OR = 0.92, 95% CI: 0.60–1.43, Figure 4G), case-control study (OR = 0.99, 95% CI: 0.89–1.11, Figure 4G), male (OR = 1.30, 95% CI: 1.02–1.66, Figure 5G), female (OR = 0.79, 95% CI: 0.61–1.01, Figure 5G). Retinol appeared to play a protective role in women, reducing CRC risk by 21%, although there was no significant association. For men, retinol intake was significantly positively associated with the risk of CRC.

### Association of Serum Retinol and Carotenoid Levels With Colorectal Cancer Risk

**FIGURE 6:**
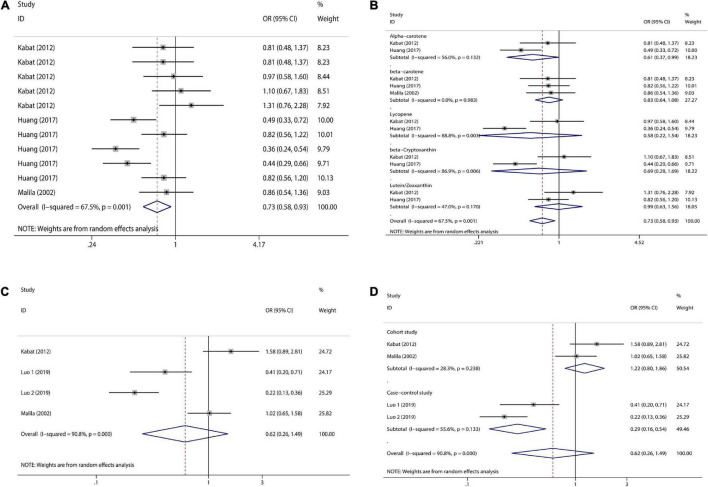
Forest plot of serum carotenoid and retinol concentrations and colorectal cancer risk. **(A)** serum carotenoid; **(B)** subgroup analysis of serum carotenoids according to their types; **(C)** serum retinol; **(D)** subgroup analysis of serum retinol by study type.

With regard to serum carotenoids, three studies were included and a total of 11 sets of data were combined. Serum total carotenoids (OR = 0.73, 95% CI: 0.58–0.93, Figure 6A) were significantly (*p*_*t*_ = 0.01) negatively associated with CRC risk. The results showed significant heterogeneity (*I*^2^ = 67.5%, *p* = 0.001), which was combined using the random-effects model. The subgroup analysis was performed according to the type of nutrients. Serum α-carotene (OR = 0.61, 95% CI: 0.37–0.99, Figure 6B) was significantly inversely associated with CRC risk. However, the serum content of β-carotene (OR = 0.83, 95% CI: 0.64–1.08, Figure 6B), Lycopene (OR = 0.58, 95% CI: 0.22–1.54, Figure 6B), and β-Cryptoxanthin (OR = 0.69, 95% CI: 0.28–1.69, Figure 6B), although negatively correlated with CRC risk, was not significant. There was no correlation between serum Lutein/Zeaxanthin (OR = 0.99, 95% CI: 0.63–1.56, Figure 6B) content and CRC risk.

With regard to serum retinol, three studies were included and a total of four sets of data were combined. High serum retinol (OR = 0.62, 95% CI: 0.26–1.49, Figure 6C) was inversely associated with CRC risk, but the association was not significant (*p*_*t*_ = 0.284). The results showed significant heterogeneity (*I*^2^ = 90.8%, *p* = 0.000), and random effects model was used for combination. Subgroup analysis were also performed according to study type. Cohort studies (OR = 1.22, 95% CI: 0.80–1.86, Figure 6D) showed no association between serum retinol and CRC risk, but case-control studies (OR = 0.29, 95% CI: 0.16–0.54, Figure 6D) showed a significant inverse association between serum retinol and CRC risk. Meta-analysis results of the above various nutrients are shown in Table 2.

**TABLE 2:** Meta-results on intake of various nutrients and colorectal cancer risk.

						Heterogeneity		
Nutrient type	Studies (*n*)	OR	95%CI	*P*-value	Model	Chi^2^	*I* ^2^	*P*-value
β-carotene	20	0.89	0.78–1.03	0.113	Random	51.61	63.2%	0.000
α-carotene	10	0.87	0.72–1.03	0.110	Random	20.14	55.3%	0.017
Lycopene	13	0.93	0.81–1.07	0.329	Random	34.83	65.6%	0.000
Lutein/zeaxanthin	12	0.96	0.87–1.07	0.508	Fix	12.31	10.6%	0.341
β-Cryptoxanthin	9	0.70	0.48–1.01	0.058	Random	58.36	86.3%	0.000
Carotenoids	19	0.97	0.81–1.15	0.717	Random	58.44	69.2%	0.000
Retinol	15	0.99	0.89–1.10	0.850	Fix	21.37	34.5%	0.092
Carotenoids (serum)	11	0.73	0.58–0.93	0.010	Random	30.79	67.5%	0.001
Retinol (serum)	4	0.62	0.26–1.49	0.284	Random	30.51	90.8%	0.000

### Publication Bias and Sensitivity Analysis

**FIGURE 7:**
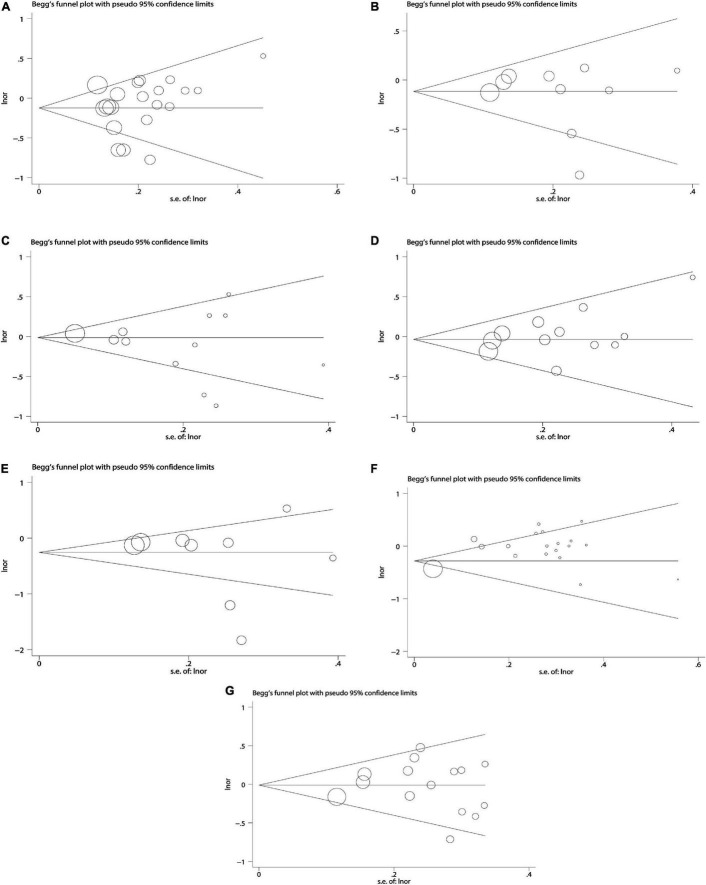
Begg’ s publication bias plots on dietary carotenoids and retinol and colorectal cancer risk. **(A)** β-carotene; **(B)** α-carotene; **(C)** lycopene; **(D)** lutein/zeaxanthin; **(E)** β-cryptoxanthin; **(F)** carotenoids; **(G)** retinol.

**FIGURE 8:**
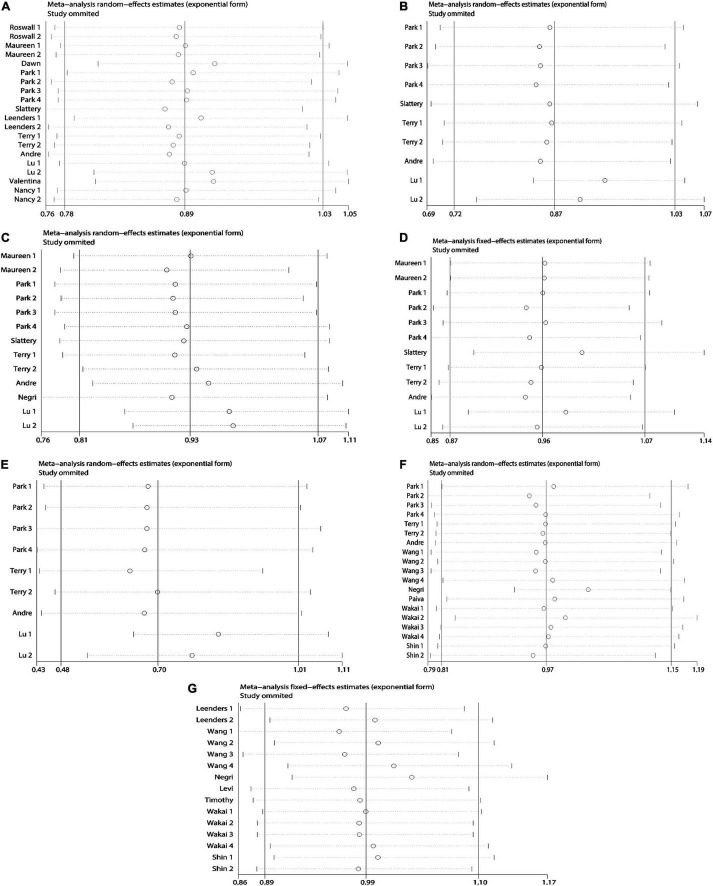
Sensitivity analysis plots on dietary carotenoids and retinol and colorectal cancer risk. **(A)** β-carotene; **(B)** α-carotene; **(C)** lycopene; **(D)** lutein/zeaxanthin; **(E)** β-cryptoxanthin; **(F)** carotenoids; **(G)** retinol.

Due to the less in serological studies included, bias testing and sensitivity analysis were not necessary. Therefore, we performed bias test and sensitivity analysis on the combined results of dietary retinol and carotenoids. We used Begg’s test as well as Begg’s funnel plot to assess publication bias. Begg’s test results (Figure 7): β-carotene (Pr > | z | = 0.417), α-carotene (Pr > | z | = 0.721), lycopene (Pr > | z | = 0.464), β-Cryptoxanthin (Pr > | z | = 0.075), Lutein/Zeaxanthin (Pr > | z | = 0.304), Carotenoids (Pr > | z | = 0.234), retinol (Pr > | z | = 0.692). The results of bias test showed that all funnel plots were symmetrical and (Pr > | z | > 0.05), indicating that no significant publication bias was found in the combined results. Sensitivity analysis (Figure 8) of the results was performed and the pooled OR varied in a limited range without significant change after removing each study, indicating that our results were stable. From this, it can be seen that the relevant conclusions we draw are stable and reliable.

## Discussion

Although vitamin A (retinol) and carotenoids are widely present in a variety of vegetables and fruits, many people still lack the intake of these nutrients. Therefore, the impact of retinol and carotenoids intake on CRC risk has important public health implications. We included a total of 22 studies that pooled clinical studies on dietary and serum retinol and carotenoids and CRC risk. Subgroup analysis was performed according to tumor type, study category and sex.

The results showed that dietary β-carotene intake was not significantly negatively correlated with CRC risk, but β-carotene could significantly lower CRC risk in the male population, showing a protective and preventive effect. The consumption of α-carotene lowered the risk of CRC, however, the link was not statistically significant. A high lycopene consumption lowered CRC risk marginally but not dramatically. There was no link seen between high lutein/zeaxanthin consumption and CRC risk. High intake of β-cryptoxanthin was able to non-significantly reduce the risk of CRC. There was no link found between high total carotenoids consumption and CRC risk. High retinol consumption had no significant connection with CRC risk, and although it appeared to protect women and reduce CRC risk by 21%, high retinol intake was able to significantly increase the risk of CRC for men, and this difference was very important and could guide dietary matching. In summary, high β-carotene, α-carotene, lycopene, and β-cryptoxanthin all have a tendency to reduce CRC risk, which is more pronounced in the male population, but there is uncertainty that must be explored. Total carotenoids intake and Lutein/Zeaxanthin intake were not associated with CRC risk. Beta-carotene has a preventive effect on CRC in men and retinol seems to have a preventive effect in women and a carcinogenic effect in men, and this difference has led us to new ideas about adjusting the diet by sex.

In addition to focusing on the risk of dietary retinol and carotenoids on CRC, we also focused on serological aspects of the study. A significant inverse association was found between serum carotenoids concentrations and CRC risk. Serum β-carotene was shown to have a substantial negative relationship with CRC risk. Other carotenoids, while adversely associated, were not significant. Case-control studies have found a substantial negative link between serum retinol and CRC risk, while cohort studies have found no significant relationship, hence the relationship between serum retinol and CRC remains unknown and must be confirmed by large prospective investigations. Most previous studies have focused on dietary carotenoids, but in recent years attention has gradually shifted to serum carotenoids, possibly due to the development of serum detection techniques and more stable and accurate quantitative assessment of serum. Serum carotenoids are widely studied, in addition to CRC (28, 29), but also associated with the risk of breast cancer (30), lung cancer (31), prostate cancer (32, 33) and hepatocellular carcinoma (34), which can be used as a key research direction in the future. If the relationship between serum carotenoids and various cancers can be clearly understood, routine admission examination can be performed in high-risk cancer population to preliminarily evaluate and screen related tumors, which has certain application prospects in clinical practice.

We will further explore the mechanisms underlying the prevention and suppression of CRC by carotenoids. β-carotene has been found in animal studies to have anti-colon cancer properties through modulating M_2_ macrophages and activated fibroblasts (35). By regulating K-ras, PKB, and beta-catenin, dietary lutein can decrease colon carcinogenesis caused by p-dimethylhydrazine in rats (36). Carotenoids isolated from Chlorella ellipsoidea and Chlorella vulgaris have also been shown in cell tests to have antiproliferative and anticancer effects on human colon cancer cells (37). B-carotene has been demonstrated to decrease colon cancer cell development by reducing COX-2 production and down-regulating colon cancer cell homeostasis (38). A growing number of experimental investigations have also proven the mechanism and significance of carotenoids in anti-CRC. Our study surprised us by the finding that a high intake of β-cryptoxanthin (OR = 0.70, 95% CI: 0.48–1.01) was able to reduce CRC risk. β-cryptoxanthin is one of the six primary carotenoids. It is mostly present in citrus fruits, although it is also found in corn, peas, and other yellow animal products (16, 39). β-cryptoxanthin has been demonstrated in animal experiments to have preventative and inhibitory effects on a number of malignancies, including colon cancer (40), gastric cancer (41), lung cancer (42–44), bladder cancer (45), and liver cancer (46) through a variety of molecular mechanisms. It has been demonstrated that β-cryptoxanthin in combination with oxaliplatin dramatically increased the apoptosis of colon cancer cells *in vitro* and *in vivo*, indicating anti-tumor and therapeutic actions on CRC (40). From this point of view, although there are few studies on β-cryptoxanthin, it may have a role in preventing and inhibiting tumors in a variety of cancers, especially CRC. The conclusions about β-cryptoxanthin in this meta-analysis should be paid attention to, and strengthening the study of β-cryptoxanthin may bring fruitful results.

There have also been several earlier meta-analyses investigating the relationship between carotenoids and CRC. Männistö et al. performed a meta-analysis of cohort studies on dietary carotenoids and CRC risk in 2006, and discovered no link between any carotenoids and CRC risk (47). Conclusion may be caused by several limitations. On the one hand, we believe that the relevant studies it includes are somewhat old and not suitable for the dietary pattern of modern humans. On the other hand, the studies it included were Caucasian studies in Europe and North America with certain geographical limitations; at the time, communication technology was limited, which easily led to a loss due to follow-up bias. Wang et al. performed a meta-analysis of observational data on lycopene consumption and CRC risk in 2016 (48). The data indicate that lycopene consumption is not related with an increased risk of CRC, which is consistent with our findings. In 2016, Panic et al. performed a meta-analysis of dietary carotenoid consumption and CRC risk, which found no significant link between dietary carotenoid intake and CRC (49). The reason for the inconsistency with our findings is that on the one hand we updated and added several new studies, on the other hand our study was performed in strict accordance with the quality assessment rules and removed several unqualified studies, and his study included these low-quality articles, which may affect the results. Third, we also conducted a gender subgroup analysis that may derive the effect of gender differences, and his study did not consider gender differences. Our findings are not consistent with the above the meta-analyses, but our study is higher credible.

We found clear heterogeneity in the entire summary results for retinol and carotenoids and CRC risk. Heterogeneity is inevitable in meta-analysis, and determining the source of the heterogeneity is an important step. First, where the heterogeneity of the data was considerable, we utilized a random-effects model to combine effect sizes. Second, we conducted a subgroup analysis by tumor type, research type, and gender. Most studies’ heterogeneity was greatly decreased after subgroup analysis. Third, we conducted a sensitivity analysis to exclude the one that had the biggest influence on the research outcomes, hence lowering heterogeneity. In addition, there may be many factors that can increase heterogeneity, such as differences in race, region, dietary structure, ideology, and degree of economic development. Finally, heterogeneity may ensue as a result of non-uniform methodologies and research details, as well as inconsistency between meals and vitamin A content measurement instruments or scales. Heterogeneity is exacerbated by the inconsistency of particular dosage limits for high and low intakes. As a result, the conclusions drawn should be treated with caution.

Our meta-analysis provides a number of advantages. First, for the first time, we not only evaluated the association between dietary carotenoids and retinol and CRC risk, but also performed serological aspects. Furthermore, each of the six major groups of carotenoids was thoroughly examined. Second, because this study included a large number of cases and participants, more reliable estimations of the connection between retinol and carotenoids consumption and CRC risk may be obtained. Third, there was no evidence of significant publication bias in our meta-analysis. Fourth, we conducted a detailed subgroup analysis according to tumor type, study type, and gender. Fifth, the results of our included studies were all adjusted for covariates. Sixth, we included studies from the last 20 years, avoiding that old dietary patterns influence the accuracy of study conclusions.

Our study has several limitations. First, we only included English articles, which may cause selection bias. Second, there is a large heterogeneity in the findings, although the sources of heterogeneity have been explored. Third, study results were not subgroup analyzed by region and race. Fourth, the specific doses of retinol and carotenoids were not stated, and no dose-response meta-analysis was performed. Fifth, detection and transformation tools for retinol and carotenoids contained in ingested foods are not described. Sixth, although all results were adjusted for covariates, it is possible that there are other factors that affect the accuracy of the results.

While the efficacy of early CRC has improved, the prognosis of advanced CRC remains poor, so we must invest more effort in cancer prevention. Through the transformation of scientific research achievements, develop a set of preventive means suitable for the CRC population, strengthen people’s health publicity and education from the aspects of diet, exercise, and mental psychology, and eliminate tumors in the bud. From the results of our study, it can be seen that retinol, β-cryptoxanthin, β-carotene, α-carotene, and lycopene have some value in preventing CRC. We suggest that middle-aged and older adults with a family history of CRC or other risk factors can prevent CRC by modestly increasing carotenoid intake and even by taking supplements. From our gender subgroup analysis, it can be seen that β-carotene has a preventive effect on CRC in men, while retinol seems to have a preventive effect on CRC in women, so we can develop corresponding dietary recipes according to gender, and agents with different allocation ratios can also be made when designing supplement components to make cancer prevention more accurate.

Internationally, most of the research on vitamin prevention of cancer stays at the level of observational studies, and most of them have not been studied more deeply. In the future, on the one hand, perfect inclusion and exclusion criteria can be developed to conduct multicenter large randomized controlled trials (RCTs) to clarify the preventive effect. On the other hand, rigorous animal experiments and tumor cell experiments can be designed to determine the preventive effect, as well as to clarify the preventive mechanism. If validated by multicenter RCT and cell and animal experiments, recipes for relevant populations can be developed and corresponding supplements can be manufactured for promotion and application. Vitamins are one of the essential nutrients for human beings, which contain a wide variety and have different physiological functions and have an important relationship with many diseases. While exploring retinol and carotenoids, we can also try to explore the preventive effects of vitamin B, vitamin C, vitamin D, vitamin E, and folic acid on different cancers. In addition to exploring the value of vitamins in the prevention of cancer, the value of survival and prognosis was explored. In addition, we found that most of the vitamins belong to antioxidants, and in addition to studying the value of vitamins in cancer, the relationship between other antioxidants and cancer can be explored, such as melatonin, anthocyanins, astaxanthin, and quercetin. Finally, it should be noted that excessive use of vitamins will produce corresponding toxic side effects, such as excessive intake of carotenoids will cause loss of appetite, yellow skin, poor sleep, affecting female ovulation and so on (50–52). Therefore, we recommend an appropriate increase in vitamin A intake within a safe dose range, especially an increase in dietary intake of vegetables, fruits, and animal products.

## Conclusion

Total carotenoids intake and Lutein/Zeaxanthin intake were not associated with CRC risk. High β-carotene, α-carotene, lycopene, and β-cryptoxanthin all tended to reduce CRC risk, with a more pronounced effect in the male population. In addition, β-carotene had a significant preventive effect on CRC in men. In the female population, high dietary retinol intake can reduce CRC risk, while it has carcinogenic effects in men. On the other hand, serum carotenoids concentrations were significantly and inversely associated with CRC risk. Finally, due to the limitations, large prospective studies with adequate sample size, well-controlled confounders, and long-term follow-up are needed for further exploration.

## Data Availability Statement

The original contributions presented in this study are included in the article/Supplementary Material, further inquiries can be directed to the corresponding author/s.

## Author Contributions

XH and RZ conceived the study and wrote the draft. GZ and YJ performed the literature search. DW and YW extracted the required data. XH performed the statistical analyses. HC reviewed the manuscript. All authors viewed and gave permission to publish this manuscript.

## Conflict of Interest

The authors declare that the research was conducted in the absence of any commercial or financial relationships that could be construed as a potential conflict of interest.

## Publisher’s Note

All claims expressed in this article are solely those of the authors and do not necessarily represent those of their affiliated organizations, or those of the publisher, the editors and the reviewers. Any product that may be evaluated in this article, or claim that may be made by its manufacturer, is not guaranteed or endorsed by the publisher.
